# Inhibiting Heat Shock Factor 1 in Human Cancer Cells with a Potent RNA Aptamer

**DOI:** 10.1371/journal.pone.0096330

**Published:** 2014-05-06

**Authors:** H. Hans Salamanca, Marc A. Antonyak, Richard A. Cerione, Hua Shi, John T. Lis

**Affiliations:** 1 Department of Molecular Biology and Genetics, Cornell University, Ithaca, New York, United States of America; 2 College of Medicine, Upstate Medical University, Syracuse, New York, United States of America; 3 Department of Molecular Medicine, College of Veterinary Medicine, Cornell University, Ithaca, New York, United States of America; 4 Department of Biological Sciences and the RNA Institute, University at Albany, State University of New York, Albany, New York, United States of America; 5 Department of Biomedical Engineering, Dongguk University, Seoul, South Korea; University of South Florida, United States of America

## Abstract

Heat shock factor 1 (HSF1) is a master regulator that coordinates chaperone protein expression to enhance cellular survival in the face of heat stress. In cancer cells, HSF1 drives a transcriptional program distinct from heat shock to promote metastasis and cell survival. Its strong association with the malignant phenotype implies that HSF1 antagonists may have general and effective utilities in cancer therapy. For this purpose, we had identified an avid RNA aptamer for HSF1 that is portable among different model organisms. Extending our previous work in yeast and Drosophila, here we report the activity of this aptamer in human cancer cell lines. When delivered into cells using a synthetic gene and strong promoter, this aptamer was able to prevent HSF1 from binding to its DNA regulation elements. At the cellular level, expression of this aptamer induced apoptosis and abolished the colony-forming capability of cancer cells. At the molecular level, it reduced chaperones and attenuated the activation of the MAPK signaling pathway. Collectively, these data demonstrate the advantage of aptamers in drug target validation and support the hypothesis that HSF1 DNA binding activity is a potential target for controlling oncogenic transformation and neoplastic growth.

## Introduction

The Heat Shock Factor 1 (HSF1) is a transcription factor that responds to a variety of environmental stressors to activate the heat shock response in eukaryotes, a protective mechanism conserved among different kingdoms [1]. Stressful insults, such as thermal exposure, stimulate HSF1 to act as a master activator of a set of target genes. In particular, it causes the accumulation of proteins with chaperoning activities, such as heat shock proteins (HSP), HSP70 and HSP90, which help maintain intracellular homeostasis by guarding the proteome against the toxic effects of protein misfolding and aggregation [2]. While there is only one HSF in *Saccharomyces cerevisiae* and *Drosophila melanogaster*, multiple isoforms exist in mammals and plants, which appear to have specialized functions [3]–[5]. HSF1 function is essential for the stress response and viability in yeast [6], and important for oogenesis and early development in Drosophila [7]. HSF1 is also involved in the aging process in *C. elegans* [8], as well as in extra-embryonic development and several important diseases in mammals [9].

Paradoxically, under certain circumstances, HSF1's ability to promote cell survival may endanger the overall well-being of a multicellular organism. A poignant example is the function of HSF1 in cancer. It has long been noted that cancer cells can continue to proliferate in hypoxic and hostile microenvironments that are otherwise growth inhibitory to normal cells. Therefore, it is not surprising that cancer cells exhibit elevated levels of heat shock proteins (HSPs) to facilitate the folding of damaged proteins and solubilization of protein aggregates [10]. While this observation suggests an indirect role of HSF1 in cancer progression, a direct impact of HSF1 on malignancy has been discovered recently [11]. Indeed, HSF1 is found to be activated in a wide variety of malignant cells, and the pattern of DNA occupancy by HSF1 in such cancer cells is different from that of normal cells exposed to heat shock. Thus, HSF1 drives a distinct transcriptional program that promotes cellular transformation and maintains malignant growth. In addition to many classical heat shock genes, cancer-specific genes in this program support cell-cycle regulation, signaling, metabolism, adhesion and translation. Because this HSF1 signature is associated with poor patient outcomes in different types of human cancers including those of breast, lung and colon, HSF1 may be a target for general and effective cancer therapeutics [11].

For the purpose of studying and controlling the function of HSF1 in cells and organisms, we previously identified an RNA aptamer, AptHSF-RA1 [12]. The aptamer was initially isolated in an *in vitro* selection experiment using Drosophila HSF1 as the target, and later shown to be able to recognize HSF1 in yeast, Drosophila and humans. Deletion analysis defined a minimal binding motif of the aptamer comprised of two stems and one stem–loop joined by a 3-way junction [12]. This aptamer interacts with the DNA binding domain and an adjacent linker region of HSF1, and competes with the heat shock DNA elements (HSEs) for binding to HSF1. In yeast cell extracts, the aptamer inhibits transcription from heat shock promoters, and when expressed in living yeast cells, it produces a temperature sensitive growth retardation phenotype and specific decrease of heat shock gene expression [13]. In Drosophila, this aptamer reduces Hsp83 levels and causes developmental abnormalities that mimic the phenotypes of Hsp83 reduction. The aptamer also effectively suppresses the phenotypes induced by constitutively active forms of the EGF receptor and Raf oncoproteins, which are regulated ‘client’ proteins of Hsp83 [14].

Here in the present study, we report the anti-cancer activity of this HSF1 aptamer in cultured human cells. We adopted the dimeric configuration of AptHSF-RA1 used in Drosophila [14], which was named iaRNA ^HSF1^ (“ia” stands for “inhibitory aptamer”), and delivered it into HeLa cervical carcinoma cells in the form of a synthetic gene by transfection. The anti-cancer activity of the aptamer was then investigated through three lines of studies. First, we confirmed the molecular mechanism of the aptamer action by determining the disruption of HSF1's interaction with its cognate DNA elements *in vitro* and *in vivo*. Second, we demonstrated the ability of the aptamer, when expressed in cancer cells, to promote apoptosis and inhibit colony formation in soft agar. Finally, we address the issue of specificity in cells by showing: (1) the aptamer phenotypes are suppressed by overexpression of HSF1 or HSPs, (2) aptamer expression reduces HSF1 target gene expression, and (3) aptamer expression reduces the HSF1 modulated, MAPK signaling pathway. Collectively, our results agree with previous reports that show that HSF1 is critical for the maintenance of cellular transformation. Moreover, our findings raise the interesting possibility that an aptamer that functionally inactivates HSF1 can be used to block human cancer progression.

## Methods and Materials

### Expression constructs

The sequence of the dimeric aptamer construct, iaRNA^HSF1^ is the following (the lowercase letters represent the hammerhead ribozyme): 5′GUCGAGUGACGUUGGCAUCGCGAUACAAAAUUAAGUUGAACGCGAGUUCUUCGGAAUUCAACUGCCUUCGUCAUACUCCUUGAAUUCAACUGCCUUCGGGCAUCGCGAUACAAAAUUAAGUUGAACGCGAGUUCUUGGAGGCUCGACguc uagcgaugugguuucgcuacugaugaguccgugaggacgaaac 3′. In the process of constructing the expression vector for the dimeric aptamer, an antisense control construct, RevRA1, was generated, using primer sets that swapped the parental and lagging strands of the aptamer coding region while keeping the hammerhead unit intact. Both sense and antisense units were cloned in gateway vectors [14] and moved into pDest51 (Invitogen) for expression of the aptamer and the control in mammalian cells. The coding sequence of “rescuing” proteins and their controls, including HSF1, HSP90, HSP70, LacZ and GFP, was each cloned downstream of the CMV promoter in a different vector with G418 as the selective marker.

### Cell culture and transfection

Cells used in this study were obtained from the ATCC and maintained according to the manufacturer instructions. HeLa (CCL-2), IMR-90 (CCL-196), 293T (CRL-3216), MCF7 (HTB-22), U87 MG (HTB-14), and BE(2)-M17 (CRL-2267) cells were grown in E-MEM low glucose medium (ATCC) supplemented with 10% FBS, 1X Pen/Strep in 5% CO_2_. MDA-MB-231 (CRM-HTB-26) were grown in RPMI media supplemented with 10% FBS, 1X Pen/Strep in 5% CO_2_. The 293T cells were grown in DMEM high glucose medium (ATCC) supplemented with 10% FBS, 1X Pen/Strep in 5% CO_2_. Upon growth to confluency, cells were trypsinized and passed into fresh medium according to ATCC instructions. These parental cells were transfected with iaRNA^HSF^ or RevRA1 RNA control-expressing vectors. Non-transfected cells were subsequently eliminated from the population by culturing the cells in 6 µg/ml blasticidin and washing the cells 24 hrs after transfection. Thereafter, cells were maintained and grown in (1 ug/ml) blasticidin. Only the blasticidin-resistant cells were used throughout subsequent assays. For the “rescue” experiments, constructs expressing human HSF1 (hHSF1), human Hsp90 (hHSP90), human Hsp70 (hHSP70), LacZ, or GFP were mixed with expression vectors for HSF aptamer (or control RNA) at a 20∶1 ratio to ensure proper and excess expression of the interested targets relative to the aptamers. After the plasmids were mixed, each mixture was transfected into HeLa cells in 6-well dishes. Non-transfected cells were washed away using methods described above and the remaining cells were maintained in selection medium.

### EMSA

The general scheme of electrophoretic mobility shift assay (EMSA) was adopted and modified from previous work [12]. RNA probes were internally labeled with [a-^32^P]UTP by using a T7 *in vitro* transcription kit (MAXIscript, Ambion, Austin, TX). The 10 µl binding solution contained 1X binding buffer, 1 µg carrier yeast RNA, 4 µg carrier BSA, 5 mM DTT, 10% glycerol, 6 units of SUPERase-In (RNase inhibitor), plus protein and labeled RNA aptamer. The concentration of the labeled RNA probe is below 1 nM in most experiments. The human HSF1 gene was obtained from the Thiele Lab [15] and was subcloned into the Gateway expression system as a His fusion. The bacterially expressed His-tagged hHSF protein was purified by Ni-NTA chromatography. This purified His-tagged hHSF1 protein was incubated with aptamer RNA at room temperature for 30 min and 10 min at 4° before loading on a 6–9% native polyacrylamide gel. The gels contained 1/4 TBE buffer and 1 mM MgCl_2_ and were run at 100–150 V at 4°C for 1–2 hr.

### RT-PCR

RT-PCR was performed 24 hours post transfection according to a protocol described previously using the following primers.

iaRNA^HSF1^ F:

5′-GTCGAGTGACGTTGGCATCG

iaRNA^HSF1^ R:

5′- GACGTCGAGCCTCCAAGAAC

iaRNA^Rev^ F:

5′-GTCGAGCCTCCAAGAACTCG

iaRNA^Rev^ R:

5′- GACGTCGAGTGACGTTGGCA

### Chromatin Immunoprecipitation (ChIP)

ChIP assay was performed 36 hours post transfection according to a protocol described previously [16] using antibodies against HSF1 or HSF2 kindly provided by Dr. Richard Morimoto.

### Western blots

Samples were prepared 72 hrs post-transfection for molecular analyses unless otherwise specified. For the examination of caspase-3 samples were prepared between 72–96 hrs. In brief, cells were fed every 48 hrs, and samples were collected by centrifugating the media, pelleting the cells. Also, adherent cells were scrapped in PBS, pelleted and combined with the cells in the media. Thereupon, samples were lysed in the presence of non-ionic detergents containing protease inhibitors, and the samples were boiled in the presence of 6X-SDS. Conventional Western blot protocols were used with the following antibodies. Primary Antibodies: G6PDH was purchased from Sigma (A9521). All other antibodies were purchased from Cell Signaling Inc.: HSF1 (No. 4356), Hsp90 (No. 4875), Hsp70 (No. 4872), Hsp60 (No. 4870), Hsp40 (No. 4868), Calnexin (No. 2433), GRP78 (No. 3177), EGFR∼p (No. 2234), Erk1/2∼p (No. 9146), total Erk1/2 (No. 9102), Caspase-3 (#9662). The PARP antibody was anti PARP-DBD, a gift from Dr. W.L. Kraus, and transglulaminase (TGM2) was a gift from Dr. R. Cerione's laboratory purchased from Thermo Scientific. Secondary antibodies were used according to proper immunoreactivity. PVDF membranes were blocked using 5% BSA and membranes were incubated primary antibodies overnight at 4°C.

### Apoptotic assay

Apoptosis was observed by quantifying the number of HeLa cells containing fragmented nuclei as visualized by DAPI staining under the microscope. In these experiments, parental, iaRNA^HSF^ or RevRA1 RNA control cells were observed for 96 hrs post transfection. In these experiments, non-transfected iaRNA^HSF^ was removed by culturing the cells in 6 µg/ml blasticidin and washing the cells 24 hrs after transfection. Only the blasticidin-resistant cells were used throughout subsequent assays. All statistical analyses in this study were calculated using student's t-test.

### Anchorage independent growth assay

6×10^3^ semi-stable transfected cells were plated on 6-well dishes in appropriate medium supplemented with 3% agarose (Type VII, Sigma A4018) over a warm layer of pre-solidified medium containing 6% agarose (Type VII, Sigma A4018). Five days after plating the cells a new 1 cm layer of media was plated over the growing cells. HeLa cells containing 17-AGG had a final concentration of 8.8 ug/dL 17-AAG agar mixture. The ability of cells to grow on soft agar was evaluated after 14 days. Colonies were analyzed under the light microscope.

## Results

### iaRNA^HSF1^ prevents HSF1 from binding to its regulatory DNA elements in HeLa carcinoma cells

**Figure 1:**
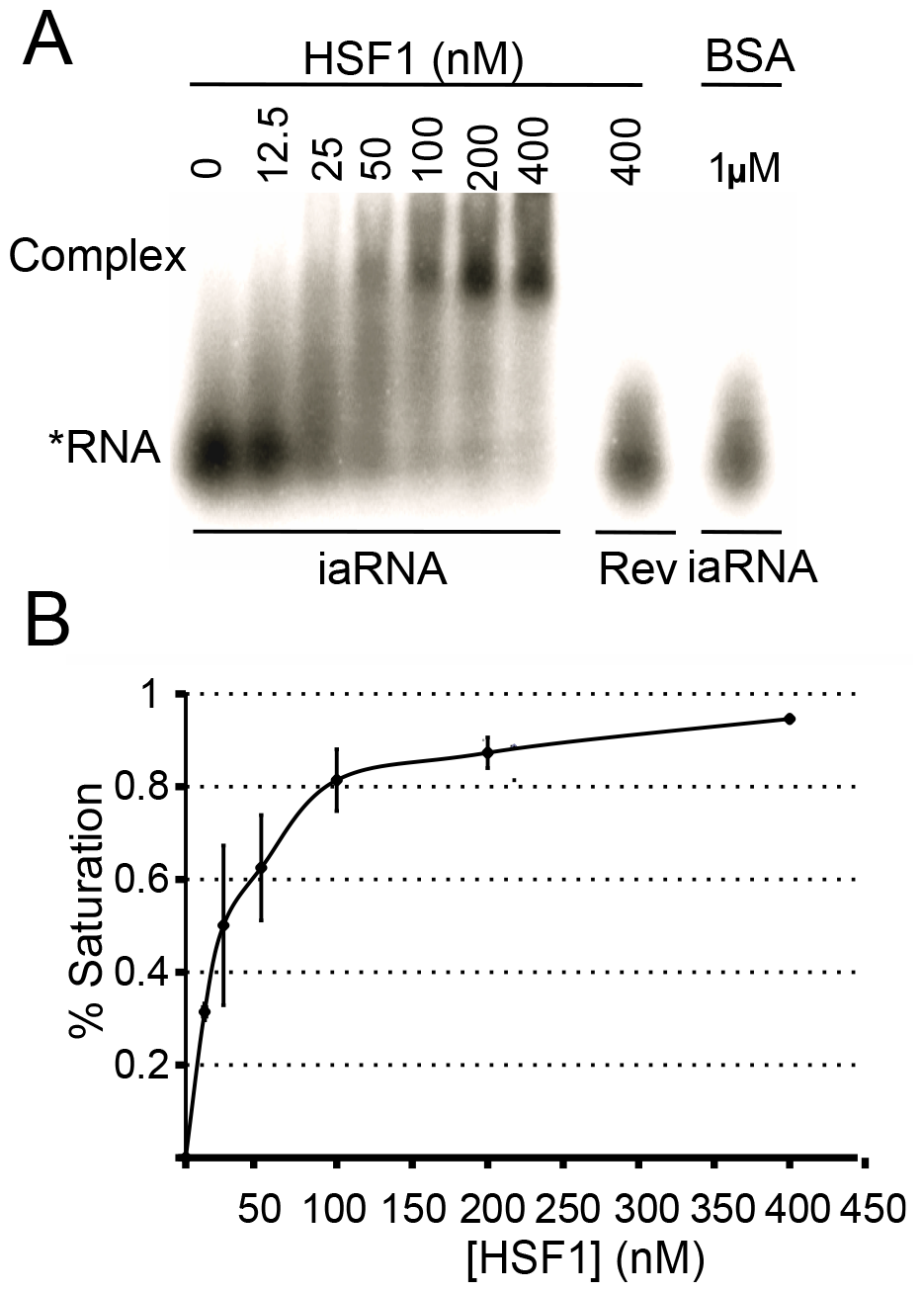
Specific binding of the aptamer to human HSF1 *in vitro*. (**A**) Electrophoretic motility shift assay (EMSA) using radiolabeled iaRNA^HSF1^ (1 nM) and increasing amounts of human HSF1 protein shows that the aptamer binds to its target avidly. (**B**) Quantification of independent EMSA reveals the apparent affinity of the iaRNA^HSF1^ for HSF1 as Kd∼25 nM (n = 5).

Previously, we developed a method for delivering RNA aptamers into the nuclei of cells as synthetic genes [17] and used it to express the aptamer for HSF1, AptHSF-RA1, in Drosophila [14]. This RNA construct, named iaRNA^HSF1^, contains two aptameric units for enhanced avidity and a hammerhead ribozyme in which the 5′ and 3′ ends of the RNA are protected to facilitate folding and stability. We adapted this construct by placing its coding sequence under the control of the EF-1a promoter in a mammalian expression vector. We also made a control construct, named RevRA1, in which the aptamer moiety of the iaRNA^HSF1^ was replaced by its antisense sequence. Before using it on cells, we produced this RNA by *in vitro* transcription and determined its avidity for purified human HSF1 in an electrophoretic mobility shift assay (EMSA) (Figure 1A) using purified human HSF1 protein (Figure S1A). Here, the iaRNA^HSF1^ generated a shifted complex with an apparent K_d_ of 25 nM (Figure 1B). In contrast, the RevRA1 control did not show any binding. In addition, when limiting amounts of iaRNA^HSF1^ was incubated with high amounts of purified BSA (1 µM), no shifted band was observed. Together, these *in vitro* results demonstrated that the interaction between iaRNA^HSF1^ and HSF1 occurs with high affinity and is relatively selective.

**Figure 2:**
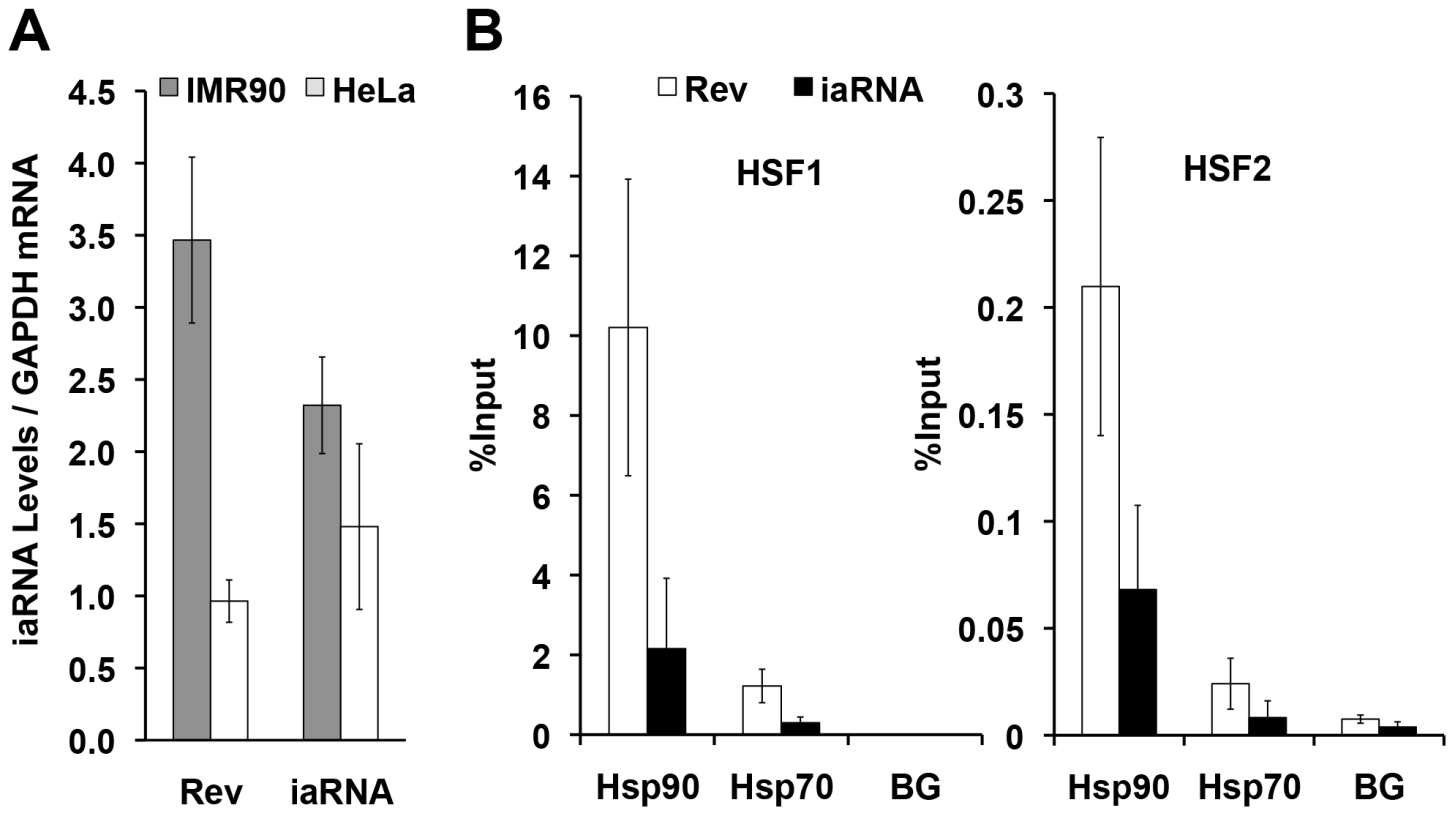
Expression of the RNA construct targeting HSF1 inhibits its occupancy at heat shock loci *in vivo*. (A) Control RNA (RevRA1) and aptamer RNA (iaRNA^HSF1^) constructs are expressed to similar levels in HeLa and IMR90 cells after 24 hrs post transfection (RNA values normalized to GAPDH, n = 3). (**B**) Disruption of HSF1's interaction with its cognate DNA elements by iaRNA ^HSF1^. ChIP assays in iaRNA^HSF1^ (or RevRA1) expressing HeLa cells show that iaRNA^HSF1^ expression can effectively inhibit HSF1 binding to the *Hsp90* and *Hsp70* promoter loci *in vivo* (n = 3). Antibodies used in ChIP assays are specific for mammalian HSF1 or HSF2 proteins [20]. BG  =  Background.

Next, we transfected the iaRNA^HSF1^-expressing vector into a cancer and a non-transformed cell line. We chose HeLa cervical carcinoma cells as a representative cancer cell line, because they are one of the most commonly used and well characterized human cancer cell lines [18]. As a non-transformed cell line, we used IMR-90 cells, which are non-transformed, non-immortalized human lung fibroblasts [19]. As a control for the functional aptamer, we used the RevRA1 expressing vector. To confirm the expression of the constructs in the cells, we performed reverse transcription and quantitative PCR (RT-qPCR) assays using primer sets that recognize the dimeric iaRNA^HSF1^ or the control RevRA1 24 hours post-transfection. Our results showed that the RNA aptamer levels were similar for both the aptamer and the control RNA in HeLa and IMR-90 cells (Figure 2A).

To confirm the molecular mechanism of aptamer action, we determined whether iaRNA^HSF1^ effectively prevented HSF1 from binding to its regulatory DNA elements in cells. For this purpose, we performed chromatin immunoprecipitation (ChIP) assays on iaRNA^HSF1^ or RevRA1 expressing HeLa cells (36 hrs post-transfection, prior to the cells undergoing apoptosis—see below) using an antibody specific to mammalian HSF1 [20]. We found that the iaRNA^HSF1^ expression significantly compromised HSF1 binding to key heat shock promoters (*Hsp90* and *Hsp70*) *in vivo* (Figure 2B). Because mammals contain two heat shock proteins, HSF1 and HSF2, and the DNA binding domain is highly conserved between them, we also tested whether our aptamer inhibits HSF2 binding. We found that iaRNA^HSF1^ expression similarly prevents HSF2 binding to the *Hsp90* and *Hsp70* promoter *in vivo* (Figure 2B). It is possible that the aptamer inhibits both transcription factors or the inhibition of one factor binding might affect the promoter occupancy of the other, as both HSF1 and HSF2 are known to regulate specific heat shock genes [21]. Nonetheless, the much lower levels of HSF2 at the promoters, relative to HSF1 (note the 50-fold difference in y-axis scale), detected by ChIP at the *Hsp90* and *Hsp70* promoters and the reduced affinity of the aptamer for HSF2 (Figure S1B) suggest that the primary inhibitory effects of the aptamer on downstream gene expression is through HSF1, at least under these conditions in HeLa cells.

### iaRNA^HSF1^ expression induces apoptosis and attenuates the transforming capabilities of cancer cells

**Figure 3:**
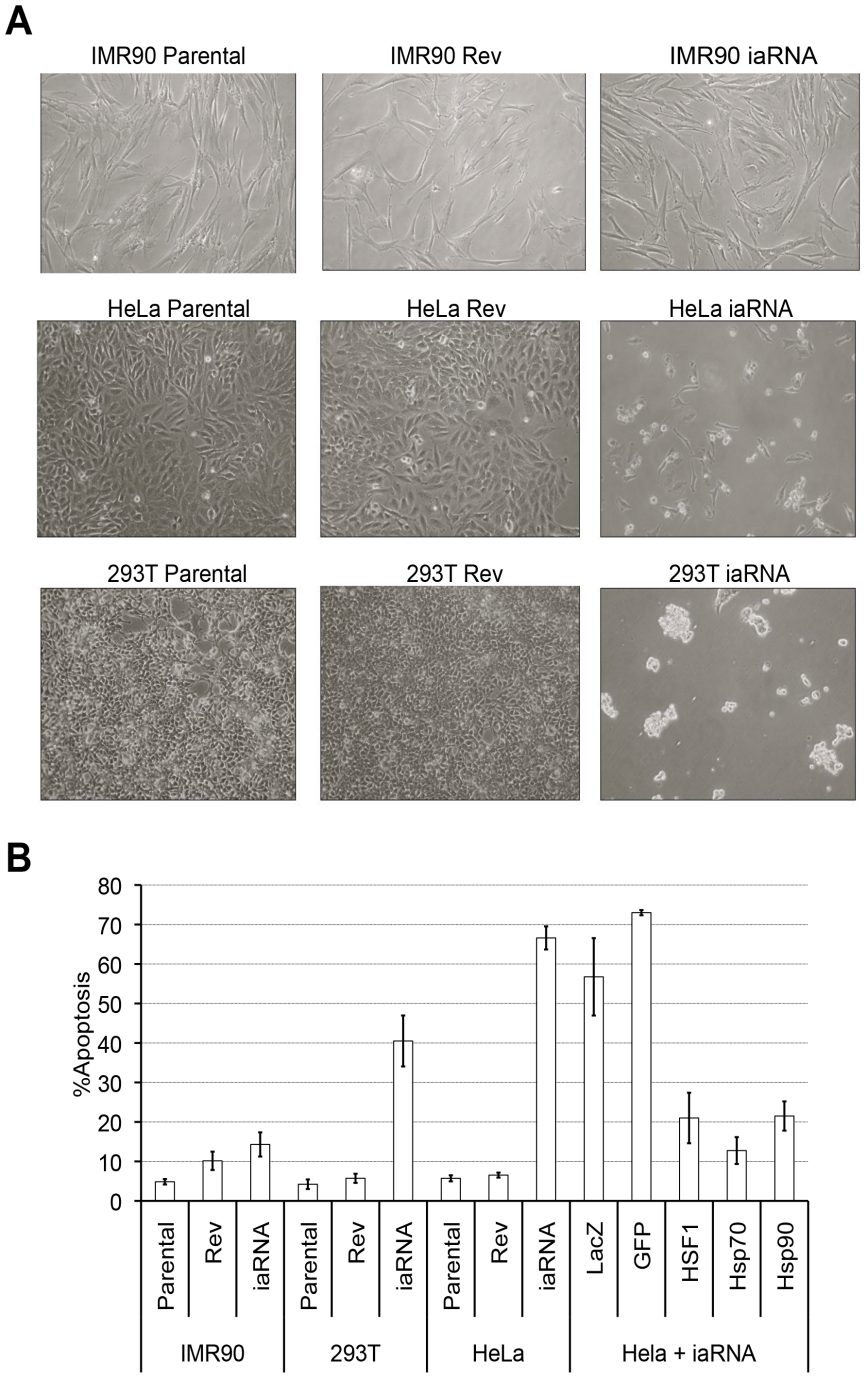
HSF1 inhibition attenuates cancer cell survival. (**A**) iaRNA^HSF1^ expression does not affect normal cells (IMR90), yet induces an abnormal morphology in HeLa cervical carcinoma and chemically transformed kidney cells (293T). (**B**). Nuclear condensation and fragmentation assays reveal that iaRNA^HSF1^ expression induces ∼10-fold increase in apoptosis in HeLa cells (p<0.0001) (n = 8), and ∼7-fold increase in apoptosis in 293T cells (p<0.0001) (n>8). Furthermore, iaRNA^HSF1^ induced apoptosis in effectively suppressed by the over-expression of molecular chaperones (HSF1 p<0.006, Hsp90 p<0.005, or Hsp70 p<0.002), but not random proteins (GFP or LacZ) (n>8).

Next, we investigated the effect that the aptamer has on the survival of HeLa cells and the non-transformed IMR-90 cells. Four days (96 hrs) after transfecting the cells with either iaRNA^HSF1^ or RevRA1, cellular viability and apoptosis was quantified by direct observation of those cells that were actively undergoing cell blebbing, nuclear breakdown and chromatic fragmentation. We found that aptamer expression in the non-transformed IMR-90 cells caused little, if any morphological changes (Figure 3A) or induction of cell death (Figure 3B). In contrast, the aptamer caused an approximately 9-fold increase in cell death of the HeLa cancer cell line (63% of the population, *p* = 0.0001). As already demonstrated above in Figure 2A, these differences were not due to differences in the levels of aptamer expression between the non-transformed and transformed cancer cell lines. To demonstrate the generality of this effect, we then asked whether expression of the iaRNA^HSF1^ in the chemically transformed human 293T kidney cell line would similarly kill the cells. We found that iaRNA^HSF1^ expression in the 293T cells dramatically causes the cells to round up and detach from their culture plates, similar to HeLa cells (Figure 3A). Compared to the parental and control cells there was an approximately 7-fold increase of apoptosis in the iaRNA^HSF1^ expressing 293T cells, with a *p*-value of 0.0018 (Figure 3B).

**Figure 4:**
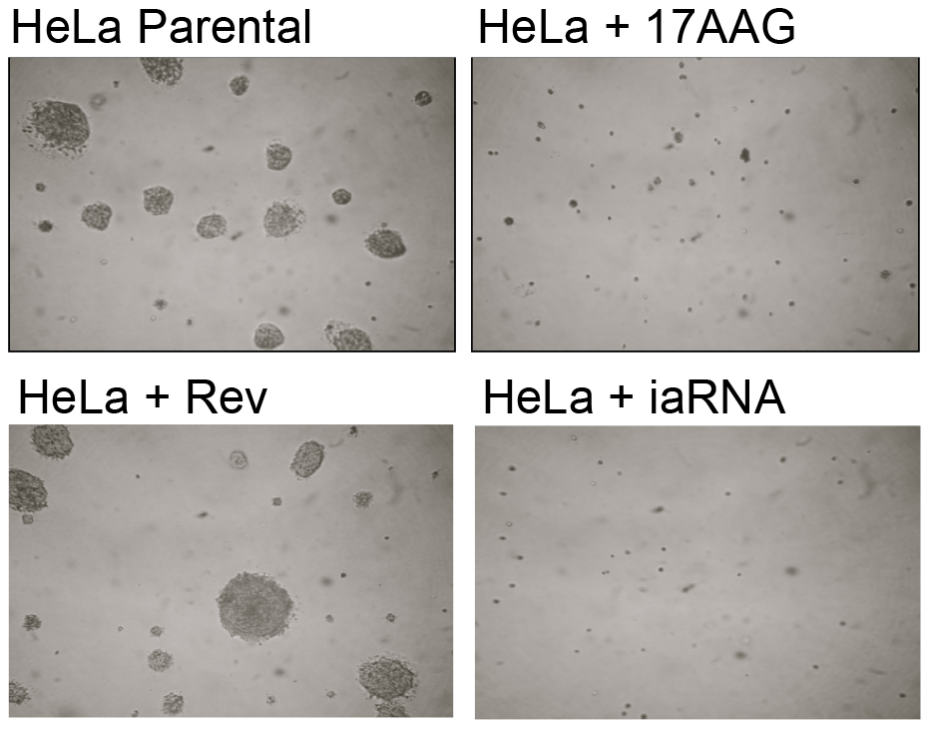
iaRNA^HSF1^ expression attenuates transformed growth. HSF1 inhibition by iaRNA^HSF1^ inhibits transformed growth in soft agar. Soft agar analysis of non-transfected HeLa cells (top left) or control RNA over-expressing HeLa (bottom left), shows that iaRNA^HSF1^ over-expression (bottom right) inhibits cellular transformation (colony formation) in a similar manner as treatment of HeLa cells with 150 nM 17-AAG (top right) (Day 14).

Since a fraction of the iaRNA^HSF1^-expressing cancer cells did not undergo apoptosis (approximately 30%), we sought to determine whether these cells were compromised in their ability to form colonies in anchorage-independent (soft agar) growth assays, an *in vitro* measurement of tumorgenicity. As expected, HeLa cells expressing a control RNA sequence (RevRA1) formed large colonies in soft agar (Figure 4); however, HeLa cells expressing iaRNA^HSF1^ did not. This indicated that the HeLa cells expressing the HSF1 aptamer that did not undergo apoptosis within the first 96 hours of its expression are compromised in their transformation capacity. These findings provide further support that HSF1 is indeed required for maintenance of the transformed cancer phenotype, similar to previous reports [22].

Because HSP90 is co-regulated by HSF1 and is important for cancer cell growth [23], we further compared the inhibitory effects of iaRNA^HSF1^ against a potent Hsp90 inhibitor 17-(Allylamino)-17-demethoxygeldanamycin (17-AAG) on colony formation in soft agar. Similarly to iaRNA expressing cells, HeLa cells grown in the presence of 150 nM 17-AAG were unable to form colonies in soft agar (Figure 4). Overall, these findings suggest that the functional inactivation of HSF1 by iaRNA^HSF1^ reduces expression of key HSF1-regulated genes that in turn are critical for maintenance of the transformed phenotype.

### iaRNA^HSF^ reduced chaperone levels and attenuated the MAPK pathway in HeLa cells

Drug target validation and therapeutic development requires rigorous assurance of specificity. This issue is pertinent to the HSF1 aptamer described here, as it was isolated using an orthologous protein. However, lack of off-target effects is difficult to prove in the complex milieu of living cells. Thus, we approached this issue from three different angles at the molecular level to demonstrate the causality between aptamer expression and the loss of the transformed phenotype in human cancer cells. First, we took a genetic approach that is similar to factor titration or add-back experiments where the aptamer induced effects are reversed by the addition of excess target protein [17]. Recently, we successfully used this strategy in Drosophila and found that the developmental abnormalities induced by the iaRNA^HSF1^ could be suppressed by the over-expression of HSF1 [14]. Following this example, we examined whether HSF1 over-expression could rescue cancer cells that express iaRNA^HSF1^ from undergoing apoptosis. We found that ectopic expression of HSF1 or HSF1-regulated genes that are important for promoting cell survival, namely, *Hsp70* and *Hsp90*, can rescue the aptamer-mediated apoptotic response, compared to the expression of control proteins (LacZ or GFP) (Figure 3B).

**Figure 5:**
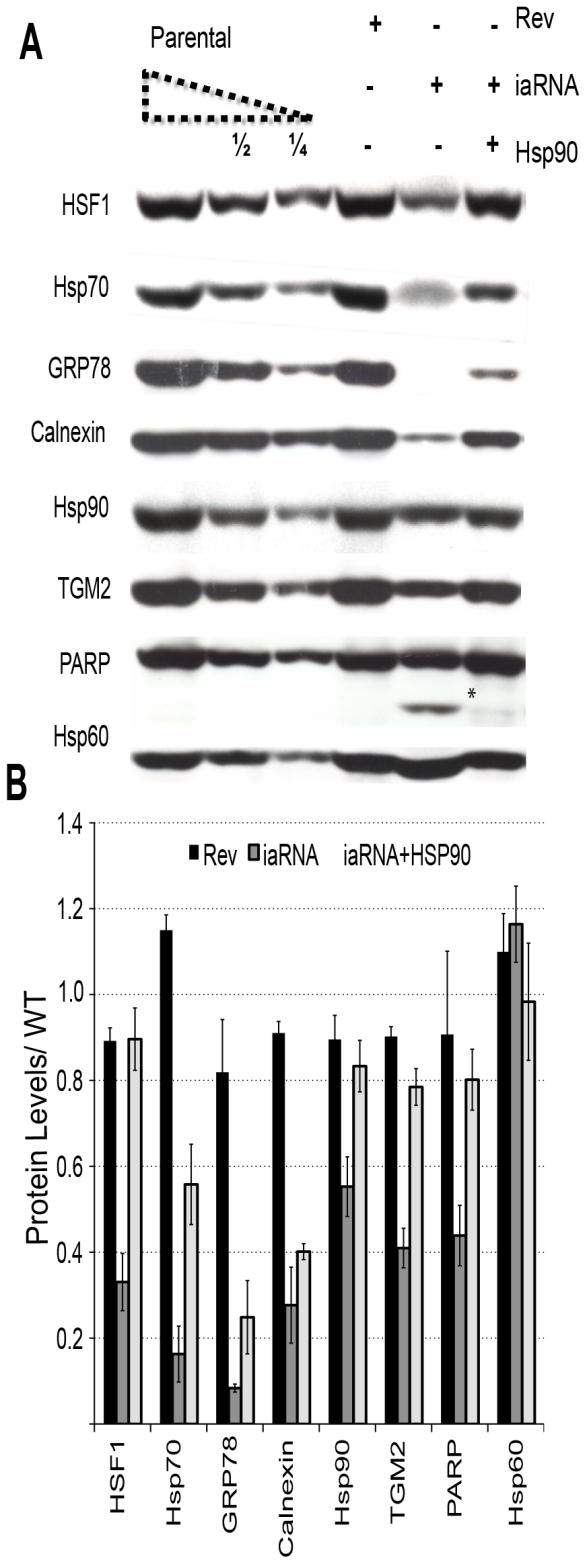
Effective targeting of HSF1 activity reduces the levels of molecular chaperone proteins. (**A**) Western blot analysis showing the depletion of specific molecular chaperone proteins in aptamer or control expressing cells. Hsp90 co-expression rescues specific molecular chaperones observed in HSF1 inhibited aptamer expressing cells. The asterisk indicates PARP degradation product, a marker of apoptosis. The left most three lanes are a serial dilution of parental line extracts that provides a quantification standard curve. (**B**) Quantification of the results observed in panel A (n = 4, error indicates %SEM).

Second, we confirmed the causality between aptamer expression and its effect on HSF1 inhibition by measuring the level of the products of genes known to be controlled by HSF1 binding, as the inhibition of HSF1 binding by the aptamer should cause a decrease in these proteins. We measured the total levels of various molecular chaperone proteins 72 hrs post transfection in parental (non-transfected), control RNA (Rev) and iaRNA^HSF1^-expressing HeLa cells (Figure 5A). Importantly, the levels of HSP70, Calnexin, transglutaminase 2 (TGM2), and Grp78 were severely decreased in aptamer expressing cells. HSP90 expression was also reproducibly decreased, though to a lesser extent than HSP70. In contrast, the level of the mitochondrial specific HSP60 chaperone was not affected by the aptamer, suggesting that HSP60 is either a highly stable protein with a long half-life that is tolerant of the proteolytic events of apoptosis or its expression is independent of HSF1 transcriptional activity. In these experiments, apoptosis was detected by assaying for the cleavage of the caspase-3 target PARP. To address whether the observed decrease in HSP70, Calnexin, transglutaminase (TGM2), and Grp78 was due to HSF1 inhibition or a consequence of the of the aptamer inducing apoptosis, we measured the total levels of chaperones in HeLa cells that expressed iaRNA^HSF^ but were prevented from entering apoptosis by co-expressing HSP90. Similar to what we observed in cells expressing only the iaRNA^HSF1^, the cells expressing both iaRNA^HSF1^ and HSP90, we observed a general decrease in the total levels of select molecular chaperones, HSP70 (∼50%), Calnexin (∼50%), and Grp78 (∼25%) compared to parental HeLa or control cells (expressing RevRA1) (Figure 5B, n = 4). This observation suggests that the reduction of molecular chaperones by iaRNA^HSF1^ is due, at least partially, to the general decrease of HSF1 activity on its gene targets.

We validate the above results and exploit the power of mammalian genetics by probing for HSP70 and HSP90 using mouse embryonic fibroblast that have been derived from viable and fertile HSF1 null mice (*hsf1−/−*) generated in Benjamin Ivor's laboratory [24]. Among mammals, HSF1 displays high structural and functional conservation [15], [20] In a similar manner as HSF1 inhibition by the aptamer, we find that *hsf1−/−* cells have relatively minor effects on the total levels of Hsp90 protein, yet displays major effects on Hsp70 under normal growth conditions when compared with wild-type MEFs (Figure S2).

Third, we examine how inhibiting HSF1 by our aptamer attenuates the activation of the MAPK signaling pathway. The Drosophila MAPK pathway was previously reported by us to be affected by the HSF1 aptamer [14]. Likewise, the Lindquist and Gius Laboratories have demonstrated that HSF1 depletion also affect this pathway [22]–[23], [25]. Here we show in human cells that HSF1 inhibition by the aptamer affected the overall levels of clinically relevant oncoproteins, Ras, Raf, and Akt, prior to the onset (48 hrs) and during aptamer induced apoptosis (96 hrs). In comparison to the RevRA1 control, the aptamer expressing cells showed a progressive decrease in the levels of Ras, Raf, and Akt in a manner that was also consistent with the decreasing levels of full-length caspase-3, a bonified marker of apoptosis [26] (Figure S3A). We find that these results can be partially suppressed by co-expression of hHSF1 both in biochemical and morphological assays (Figure S3 A&B).

**Figure 6:**
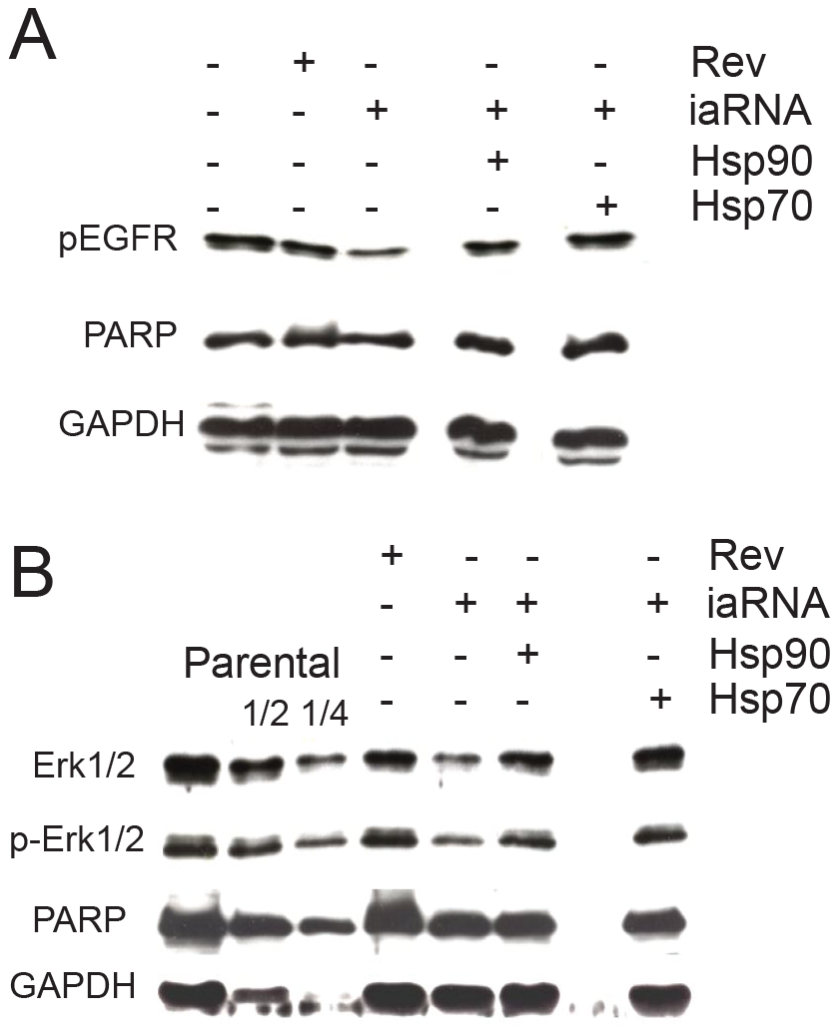
HSF1 aptamer inhibits mitogenic signaling. (**A**) HSF1 inhibition attenuates EGF receptor activation following the addition of EGF to HeLa cells. (**B**) HSF1 inhibition by iaRNA^HSF1^ causes a depletion of the total levels and activated forms of Erk1/2. The left most three lanes are a serial dilution of parental line extracts that provides a quantification standard curve. Ectopic expression of HSP70 or HSP90 suppresses the inhibition of mitogenic signaling in the iaRNA^HSF1^ expressing cells.

To directly investigate the ability of iaRNA^HSF1^ to inhibit MAPK activity in human cancer cells, we stimulated HeLa cells expressing the aptamer or the control RNA with the mitogen, epidermal growth factor (EGF), and examine the effects it had on the activation of the EGF receptor or MAPK signaling by collecting cells 10 minutes after treatment and assaying the samples by Western blotting. Importantly, these experiments were performed in a time when the cells were not actively undergoing apoptosis as visualized by the intact PARP protein (36 hrs post transfection) (Figure 6A). Overall, we find that parental or control RNA-expressing HeLa cells express a considerable amount of epidermal growth factor receptor (EGFR) that can be activated in response to epidermal growth factor stimulation (p-EGFR) (Figure 6A). In contrast, targeting HSF1 activity with the aptamer decreased the amount of activated EGFR (p-EGFR) (Figure 6A). To investigate the effect of this aptamer had on mitogenic signaling activity, we probed for activated Erk1/2 levels, members of the MAPK that function downstream of the EGFR. Here, we find that cells expressing the HSF1 aptamer contain reduced amounts of actived Erk1/2 (phosphorylated) that is accompanied by an ∼75% reduction in the total levels of Erk1/2 protein, compared to the cells expressing the control aptamer (Figure 6B). In a similar fashion and as a proof of principle, we complement these experiments using WT and *hsf1−/−* MEFs and find that HSF1 K.O. cells display a compromised ability to active Erk1/2 (p-Erk1/2) (Figure S4). This attenuated MAPK signaling response can be suppressed by over-expressing k-Ras^G12V^ in *hsf1−/−* MEF cells.

To further assess how HSF1 modulates MAPK signaling, we measured the levels of activated EGFR (phosphorylated) or Erk1/2 protein (total and phosphorylated) in aptamer-expressing cells that either over-expressed HSP90 or its co-chaperone HSP70 (36 hr post transfection). Here, over-expression of either HSP90 or HSP70 is sufficient to rescue the loss of either EGFR activation or Erk1/2 expression, resulting in an apparently normal mitogen response (Figure 6 A&B). We hypothesize that over-expression of these molecular chaperones results in the formation of a chaperone complex that enhances the stabilities of various oncoproteins that promote cancer cell survival. In this regard, we compare the effect of Hsp70 or Hsp90 inhibition in HeLa and other clinically relevant human cancer cells including brain glioblastoma (U87 MG), neuroblastoma (SK-N-BE(2)-M17), triple negative (ER-, PR-, Her2-) breast adenocarcinoma (MSA-231), and estrogen responsive (ER+, PR+, Her2−) breast adenocarcinoma (MCF7) (Figure S5). Interestingly, we find that HeLa cells and brain glioblastomas are particularly sensitive to Hsp90 inhibition, while neuroblastomas and both forms of breast adenocarcinomas are sensitive to Hsp70 inhibition. Collectively, our results suggest that HSF1 regulates specific molecular chaperones that promote oncogenic signaling and the survival of human tumors.

## Discussion

HSF1 supports highly malignant human cancers, including the most aggressive forms of breast, lung, and colon cancer [11]. It rewires the transcriptome in cancer cells, and activates the expression of not only classical heat shock proteins, but also many other genes involved in multiple processes critical for the malignant behavior of cancer. Because this HSF1-driven transcriptional program is strongly associated with metastasis and death in a wide range of cancers, HSF1 could become an effective target for cancer therapeutics [11]. However, disrupting high affinity protein-DNA interaction such as the HSF1 binding to DNA is not an easy task, and there is no current molecule or drug in commercial or clinical use that can disrupt this interaction.

The difficulty of finding small molecules to interrupt DNA-protein interaction stems from the nature of the interface. DNA binding transcription factors generally bind to an extended stretch of base pairs covering a relatively large surface area, a biophysical feature that small molecule inhibitors have a hard time competing against. In this regard, RNA aptamers have a unique advantage, as RNA molecules share quite a few common structural features with DNA. For example, several RNA aptamers have been isolated to target the DNA-binding domain of the pro-inflammatory protein NFkB [27], [28]. Structural analysis of one such NFkB aptamer revealed that the RNA was mimicking the cognate DNA element [29]. This aptamer not only inhibited the transcriptional activities of NFkB at the molecular level, but also demonstrated a potent anti-cancer activity in a mouse model [30]. In our own previous work, we isolated aptamers for the yeast TATA binding protein that are able to interrupt DNA binding and inhibit transcription [31], [32]. Similarly, the aptamer against HSF1 described here, AptHSF-RA1, binds to the DNA binding domain and adjacent linker region, effectively competing with HSE for HSF1 binding [12], [14]. These examples collectively demonstrate the capabilities of macromolecular agents in the perturbation of DNA-protein interaction.

One major challenge of macromolecular drugs is delivery. Nevertheless, before aptamers can be used in pre-clinical or clinical studies, their utility can be exploited as useful agents in drug target validation studies as demonstrated here. Currently, many drug target validation methods commonly use gene knock-out or RNAi technologies to gain insights of the underlying molecular mechanisms of disease. Unfortunately, not every protein is amenable to such inhibition, nor do these strategies provide sufficient information about the particular domains of the specific drug target in question. In recent years, we have generated a comprehensive set of data regarding the activity of the HSF1 aptamer described herein using three organisms from different biological kingdoms [12]–[14]. Because HSF1 is so highly conserved in nature, each model system has been distinctly suited and allowed us to study specific aspects of biology according to the unique experimental tractability realized by the molecular tools provided by each system. In at least two systems, we exploit the RNA aptamer technology to further understand the role of HSF1 and its regulation of molecular chaperones during development, stress, growth and survival [14].

Relevant to the case of HSF1, only a small class of naturally occurring molecules have been identified that inhibits molecular chaperones, and of these, only a handful have been tested to sensitize cancer cells to pharmacological treatment [25]. Among these, the naturally occurring anthamycin compounds like geldanamycin are better understood and have been found to inhibit Hsp90's function by binding to the ATPase domain. In recent years, geldanamycin derivatives have shown clinical benefit as seen in the phase I treatment of refractory metastatic or solid tumors [33] and the multicenter phase II trials in relapsed or refractory multiple myeloma [34]. Unfortunately, it has not yielded much benefit in the phase II advanced treatment of ovarian and peritoneal cancers [35] or the multicenter phase II trial treatment of castration-resistant prostate cancer [36]. In these refractory cases, these particular tumors might rely more heavily on other members of the HSF1 regulated chaperone network for survival. For this reason, future studies might benefit by focusing on the identification of new classes of inhibitors against the HSF1 regulated chaperone network in order to help improve the overall clinical outcomes for patients with cancer.

## Supporting Information

Figure S1 **iaRNA^HSF1^ binds avidly to human HSF1 and HSF2.** A) Bacterially expressed and His tagged full-length human HSF1 and HSF2 protein used in the *in vitro* binding assays used in this study. *In vitro* binding assays included proteins from the following bacterial preparations: HSF1a2, HSF2a6. B). Electrophoretic motility shift assay (EMSA) using radiolabeled iaRNA^HSF1^ (1 nM) and increasing amounts of human HSF2 protein shows that the aptamer binds to this target with an apparent affinity of 100–200 nM (values quantified by %shifted total complex).
(TIF)

Figure S2 **Heat shock protein profile in** ***hsf1−/−*** **mouse embryonic fibroblasts (MEF) under non-heat shock conditions (NHS).** Whole cell extracts of WT and *hsf1−/−* MEF's show that HSF1 deletion (*hsf1−/−*) results in a dramatic decrease of steady state Hsp70 protein expression levels. In contrast, the observed Hsp90 levels are only is moderately affected by HSF1 deletion, partially because this gene is under the transcriptional activity of both HSF1 & HSF2 [21]. The Hsp90 client protein, tissue transglutaminase (TGM2) displays decreased steady state levels in *hsf1−/−* MEFs. The left most three lanes are a serial dilution of parental WT extracts that provides a visual standard (n = 4).
(TIF)

Figure S3 **Suppression of iaRNA^HSF1^ effects by human HSF1 over-expression.** (A) Western blot analysis of HeLa cells demonstrates the time dependent and progressive loss of clinically significant oncoproteins (Ras, Raf, and Akt) by iaRNA^HSF1^ expression. Apoptosis can be visualized by the degradation of full-length caspase-3 starting at 48–96 hrs post transfection (lane 5 &6). (Lanes 1–3 =  serial dilutions of non-transfected parental HeLa cells at 96 hrs, lane 4 =  whole cell extract of HeLa over-expressing the RNA control at 96 hrs; lane 5 & 6 =  whole cell extract of HeLa cells over-expressing iaRNA^HSF1^ 48 & 96 hrs respectively; lane 7 =  whole cell extract of HeLa cells over-expressing iaRNA^HSF1^ & human HSF1 protein at 96 hrs). (B) Differential interference contrast imaging of HeLa cells show that human HSF1 or human Hsp90 over-expression can effectively suppress many of the morphological defects induced by iaRNA^HSF1^ expression (96 hrs) (compare this figure with samples from figure 3).
(TIF)

Figure S4 **MAPK signaling is attenuated in** ***hsf1−/−*** **MEF's.** In comparison of treatment of WT MEFs (lanes 1–3) with the potent mitogen, EGF, *hsf1−/−* cells display decreased levels of Erk1/2 activation (p-Erk1/2) (lane 4). This abnormal signaling response can be effectively suppressed by direct over-expression of constitutively activated K-Ras^G12V^ (compare lanes 4–6).
(TIF)

Figure S5 **Differential sensitivity of human cancer cells to Hsp70 or Hsp90 inhibition.** (A) Differential interference contrast imaging of HeLa, human brain glioblastoma (U87 MG), neuroblastoma (SK-N-BE(2)-M17), and triple negative (ER-, PR-, Her2-) breast adenocarcinoma (MSA-231), following Hsp70 inhibition (0.32 g/dL myricetin) or Hsp90 inhibition (8.8 ug/dL 17-AAG) (all samples taken at 72 hrs post drug treatment). (B) Quantification of nuclear condensation and DNA fragmentation (apoptotic assays) of panel (A) and estrogen responsive (ER+, PR+, Her2-) breast adenocarcinoma (MCF7) (n = 4).
(TIF)
