# Supplementary figures and images for: Inhibiting Heat Shock Factor 1 in Human Cancer Cells with a Potent RNA Aptamer
Source: PLoS One. 2014 May 6;9(5):e96330. doi: 10.1371/journal.pone.0096330 (PMC4011729; doi:10.1371/journal.pone.0096330)

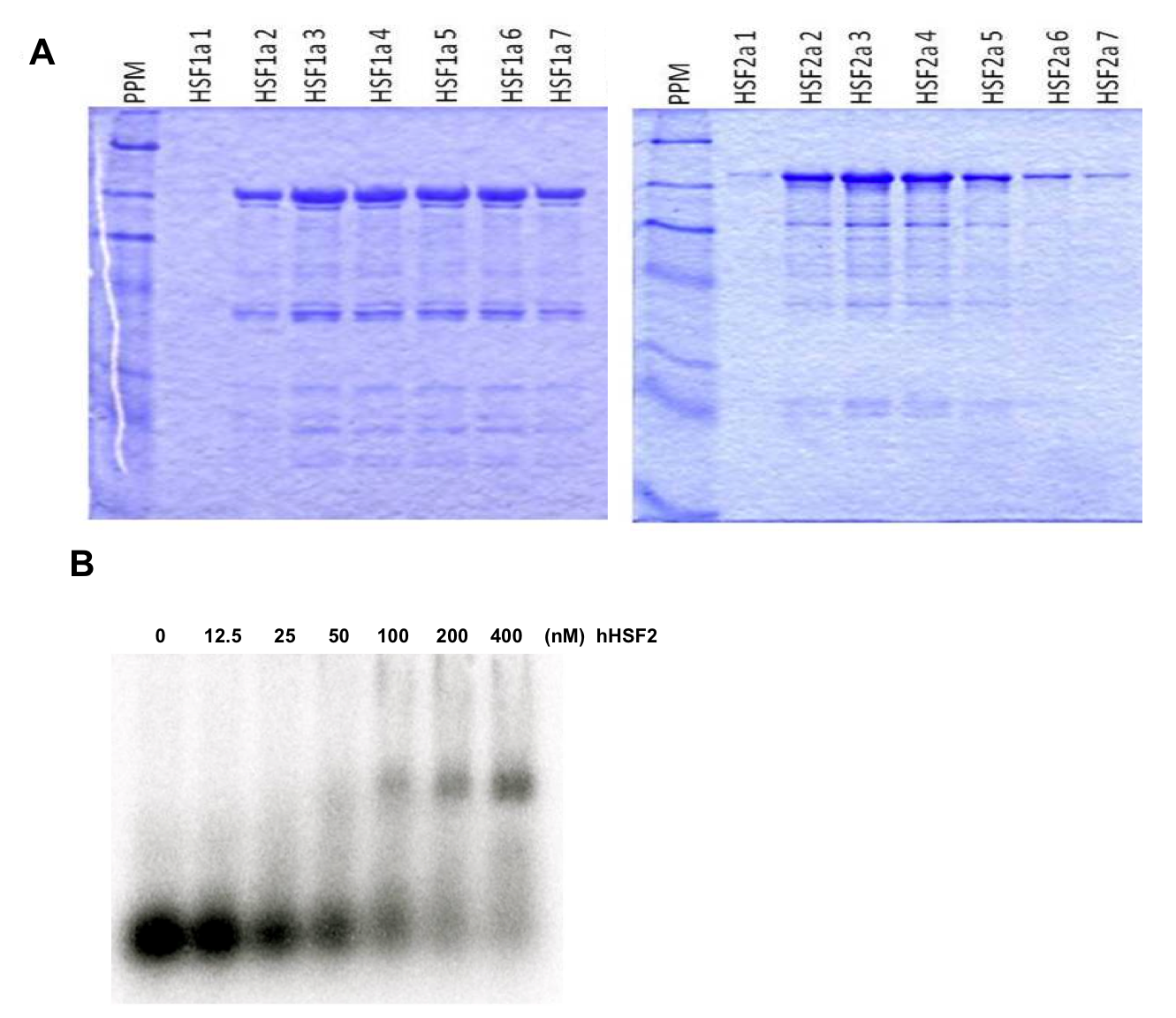

Supplement: Figure S1 — iaRNAHSF1 binds avidly to human HSF1 and HSF2. A) Bacterially expressed and His tagged full-length human HSF1 and HSF2 protein used in the in vitro binding assays used in this study. In vitro binding assays included proteins from the following bacterial preparations: HSF1a2, HSF2a6. B). Electrophoretic motility shift assay (EMSA) using radiolabeled iaRNAHSF1 (1 nM) and increasing amounts of human HSF2 protein shows that the aptamer binds to this target with an apparent affinity of 100–200 nM (values quantified by %shifted total complex). (TIF) [file pone.0096330.s001.tif]

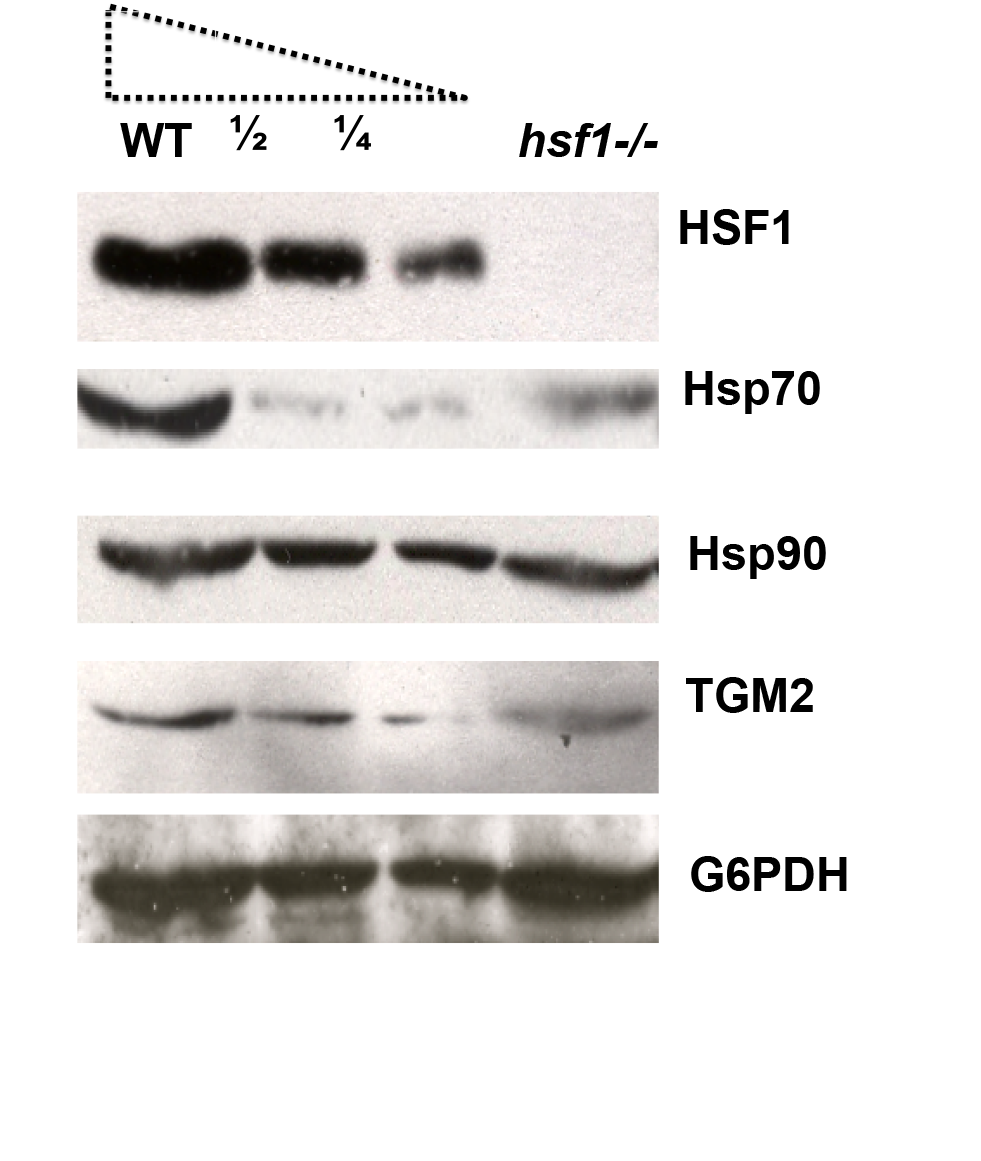

Supplement: Figure S2 — Heat shock protein profile in hsf1−/− mouse embryonic fibroblasts (MEF) under non-heat shock conditions (NHS). Whole cell extracts of WT and hsf1−/− MEF's show that HSF1 deletion (hsf1−/−) results in a dramatic decrease of steady state Hsp70 protein expression levels. In contrast, the observed Hsp90 levels are only is moderately affected by HSF1 deletion, partially because this gene is under the transcriptional activity of both HSF1 & HSF2 [21]. The Hsp90 client protein, tissue transglutaminase (TGM2) displays decreased steady state levels in hsf1−/− MEFs. The left most three lanes are a serial dilution of parental WT extracts that provides a visual standard (n = 4). (TIF) [file pone.0096330.s002.tif]

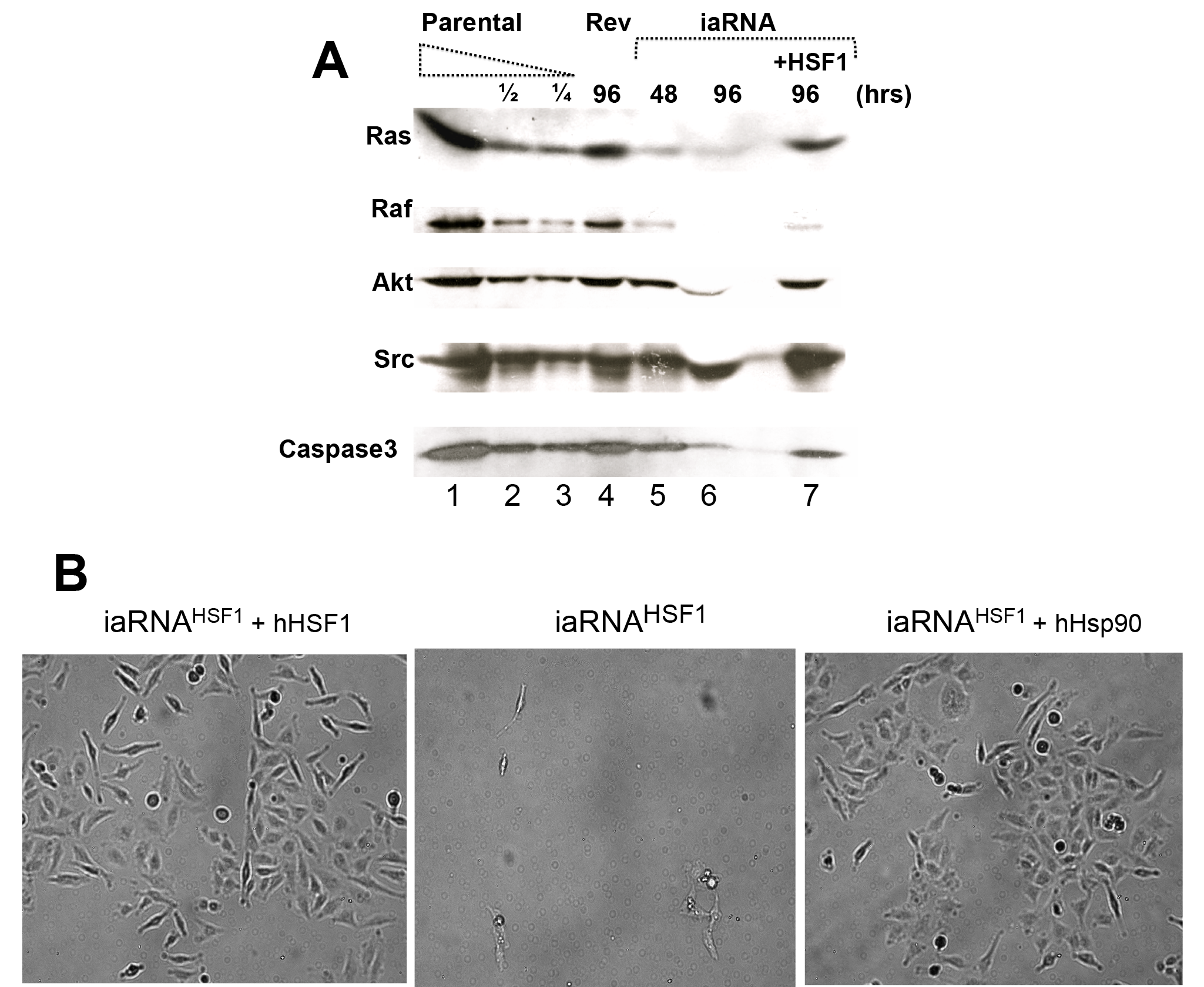

Supplement: Figure S3 — Suppression of iaRNAHSF1 effects by human HSF1 over-expression. (A) Western blot analysis of HeLa cells demonstrates the time dependent and progressive loss of clinically significant oncoproteins (Ras, Raf, and Akt) by iaRNAHSF1 expression. Apoptosis can be visualized by the degradation of full-length caspase-3 starting at 48–96 hrs post transfection (lane 5 &6). (Lanes 1–3 = serial dilutions of non-transfected parental HeLa cells at 96 hrs, lane 4 = whole cell extract of HeLa over-expressing the RNA control at 96 hrs; lane 5 & 6 = whole cell extract of HeLa cells over-expressing iaRNAHSF1 48 & 96 hrs respectively; lane 7 = whole cell extract of HeLa cells over-expressing iaRNAHSF1 & human HSF1 protein at 96 hrs). (B) Differential interference contrast imaging of HeLa cells show that human HSF1 or human Hsp90 over-expression can effectively suppress many of the morphological defects induced by iaRNAHSF1 expression (96 hrs) (compare this figure with samples from figure 3). (TIF) [file pone.0096330.s003.tif]

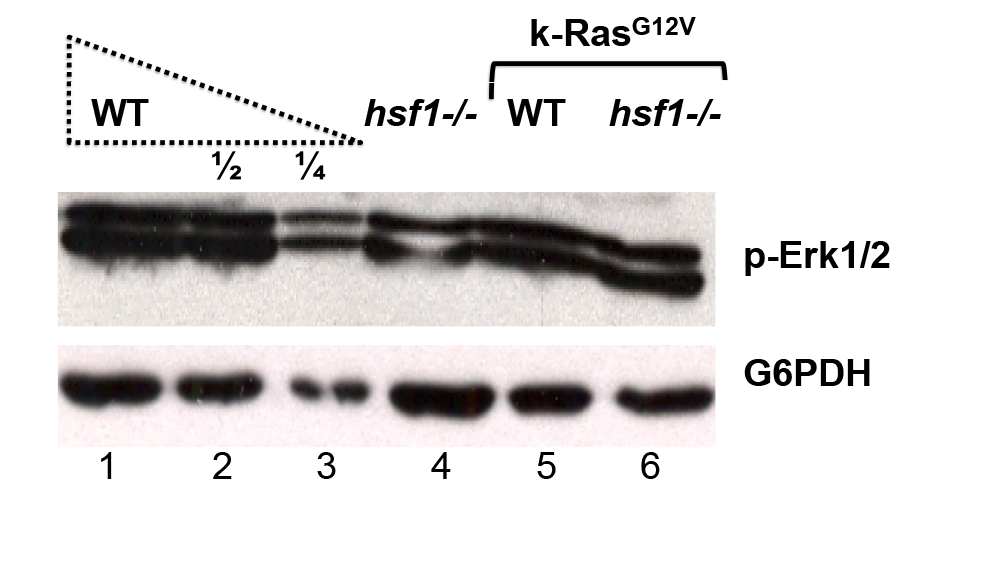

Supplement: Figure S4 — MAPK signaling is attenuated in hsf1−/− MEF's. In comparison of treatment of WT MEFs (lanes 1–3) with the potent mitogen, EGF, hsf1−/− cells display decreased levels of Erk1/2 activation (p-Erk1/2) (lane 4). This abnormal signaling response can be effectively suppressed by direct over-expression of constitutively activated K-RasG12V (compare lanes 4–6). (TIF) [file pone.0096330.s004.tif]

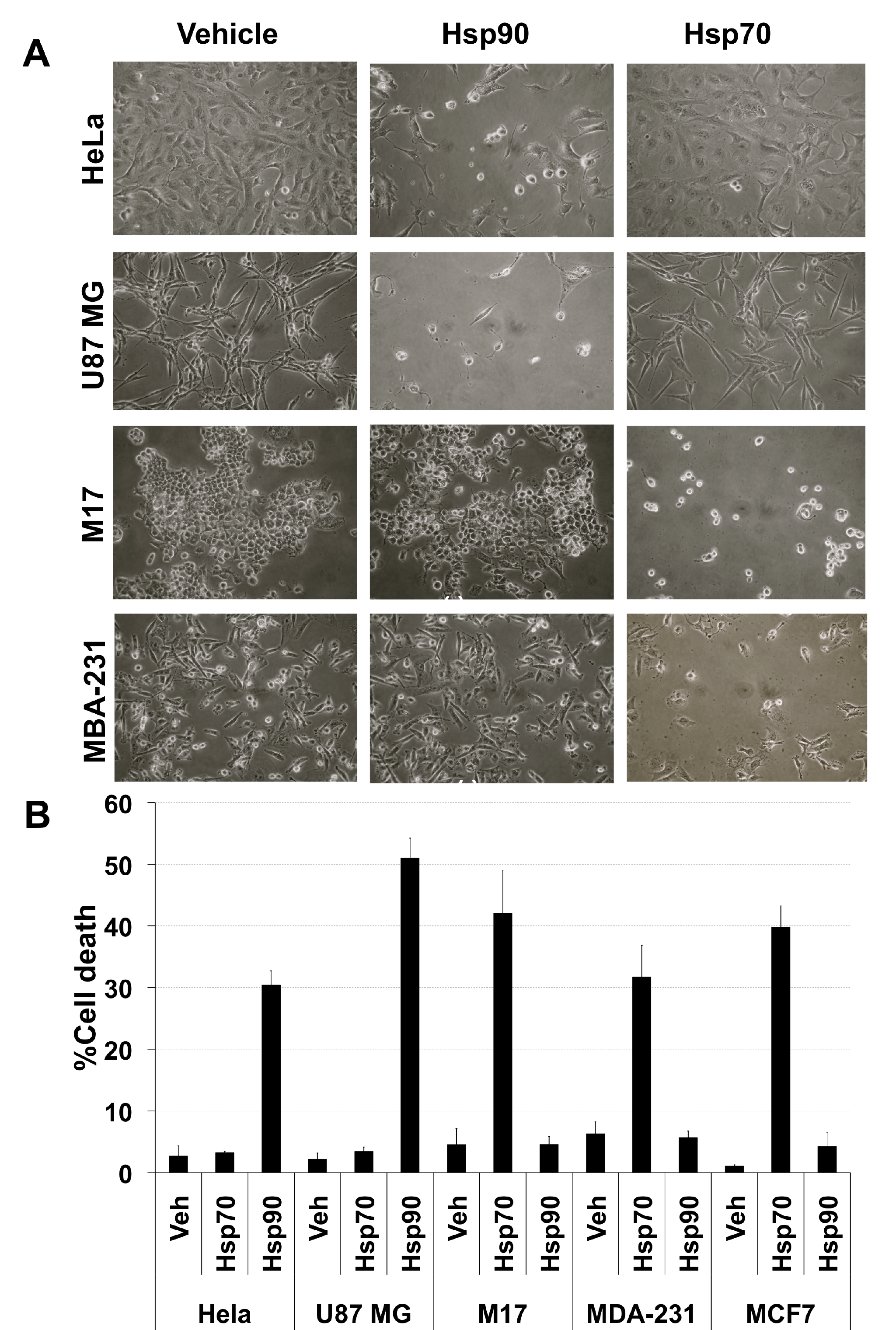

Supplement: Figure S5 — Differential sensitivity of human cancer cells to Hsp70 or Hsp90 inhibition. (A) Differential interference contrast imaging of HeLa, human brain glioblastoma (U87 MG), neuroblastoma (SK-N-BE(2)-M17), and triple negative (ER-, PR-, Her2-) breast adenocarcinoma (MSA-231), following Hsp70 inhibition (0.32 g/dL myricetin) or Hsp90 inhibition (8.8 ug/dL 17-AAG) (all samples taken at 72 hrs post drug treatment). (B) Quantification of nuclear condensation and DNA fragmentation (apoptotic assays) of panel (A) and estrogen responsive (ER+, PR+, Her2-) breast adenocarcinoma (MCF7) (n = 4). (TIF) [file pone.0096330.s005.tif]
